# Effects of Charge Traps on Hysteresis in Organic Field-Effect Transistors and Their Charge Trap Cause Analysis through Causal Inference Techniques

**DOI:** 10.3390/s23042265

**Published:** 2023-02-17

**Authors:** Somi Kim, Hochen Yoo, Jaeyoung Choi

**Affiliations:** 1 Department of Electronic Engineering, Gachon University, Seongnam-si 13120, Republic of Korea; 2 School of Computing, Gachon University, Seongnam-si 13120, Republic of Korea

**Keywords:** hysteresis, charge trapping, organic semiconductors, data analysis, causal inference

## Abstract

Hysteresis in organic field-effect transistors is attributed to the well-known bias stress effects. This is a phenomenon in which the measured drain-source current varies when sweeping the gate voltage from on to off or from off to on. Hysteresis is caused by various factors, and one of the most common is charge trapping. A charge trap is a defect that occurs in an interface state or part of a semiconductor, and it refers to an electronic state that appears distributed in the semiconductor’s energy band gap. Extensive research has been conducted recently on obtaining a better understanding of charge traps for hysteresis. However, it is still difficult to accurately measure or characterize them, and their effects on the hysteresis of organic transistors remain largely unknown. In this study, we conduct a literature survey on the hysteresis caused by charge traps from various perspectives. We first analyze the driving principle of organic transistors and introduce various types of hysteresis. Subsequently, we analyze charge traps and determine their influence on hysteresis. In particular, we analyze various estimation models for the traps and the dynamics of the hysteresis generated through these traps. Lastly, we conclude this study by explaining the causal inference approach, which is a machine learning technique typically used for current data analysis, and its implementation for the quantitative analysis of the causal relationship between the hysteresis and the traps.

## 1. Introduction

Organic semiconductors are one of the most popular alternatives to non-silicon semiconductors, owing to their ease of manufacture, mechanical flexibility, and low cost [1,2,3]. Organic field-effect transistors (OFETs) are considered very important for the development of next-generation electronic devices, owing to their advantages of low cost, large-area mass production, flexibility, lightness, and shock resistance [4,5,6,7,8,9]. Since the development of OFETs in 1980, their performance has rapidly improved based on fundamental chemical studies. Additionally, extensive research has been conducted on the effect of the implantation process and dielectric layer on semiconductor chemical structure and OFETs interface. OFETs present considerable potential since they can operate as a basic unit such as on/off switches in various electronic and optoelectronic applications [4]. Consequently, OFETs have been implemented in various applications, including printed paper, e-paper, and skin [10].

**Figure 1:**
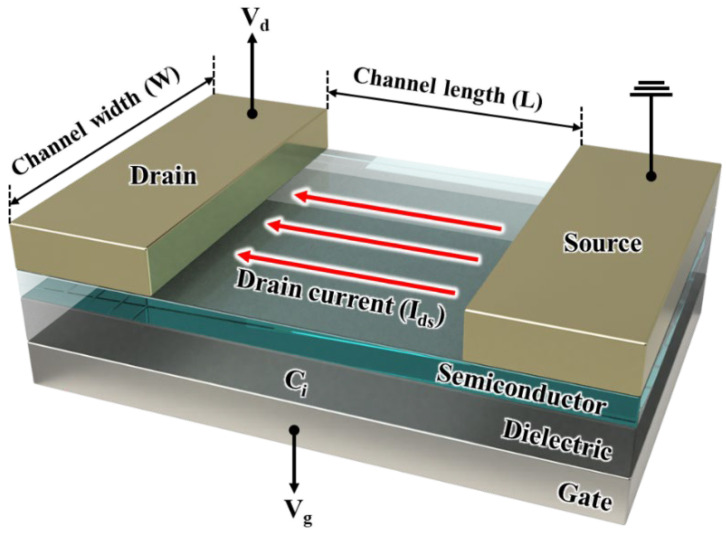
Scheme of OFET with bottom gate, top contact (BGTC).

The OFET comprises multiple electrically active layers on a substrate. These include an organic semiconductor, a dielectric, a gate, and electrodes, i.e., the source and drain electrodes, as shown in Figure 1 [11]. OFETs behave differently depending on how the potential on the gate electrode and the potential on the drain electrode behave. OFETs operate by applying appropriate voltages to three electrodes: source, drain, and gate. At this time, the current between the drain and the source is defined, $I_{d}$, and the voltages between the drain and the source, and the gate and the source are defined, $V_{d}$ and $V_{g}$, respectively. There are two types of channels: p-type and n-type. However, in an OFET, the channel is generally assumed to be p-type with hole carriers, but for n-types, it is considered that charge carriers are electron carriers. Essentially, in an ideal device, if the $V_{g}$ is zero, it is assumed that no charge has been accumulated at the semiconductor-dielectric interface. In this case, the device is considered to be off. When $V_{g}$ is applied, it causes the accumulation of charge carriers by polarizing the dielectric at the semiconductor dielectric interface. For this case, the device is considered to be on. When $V_{d}$ is applied, it causes charge carriers to accumulate at the channel, where the $I_{d}$ is measured. In the transistor channel, the charge density is modulated based on the applied $V_{g}$. However, under practical conditions, a small negative gate voltage must be first applied to fill the charge traps at the interface between the semiconductor and dielectric [4]. This trap-filling phenomenon is called the threshold voltage, $V_{t}$, and it is generated from sources such as impurities in OFETs, interfacial roughness, or some crystal defects. Additionally, due to the insignificant dopants present in the semiconductor, the threshold voltage can be positive. In this case, the device is already on, even when $V_{g}$ = 0 V, and it needs more $V_{g}$ to reach the off state [4].

There are several reasons for the occurrence of charge trapping in organic semiconductors. In particular, a significant trap density may exist at the semiconductor/gate interface in OFETs, which may deteriorate the OFET performance [10]. Devices become particularly vulnerable to carrier trapping in the case of low carrier densities since there may be injected electrons and holes that do not move, becoming local trap states. This phenomenon is modeled for density-dependent carrier mobilities; low carrier density indicates low mobility.

Charge traps are crucial in predicting and analyzing the performance of OFETs, and they can also affect the bias-stress stability with hysteresis. Furthermore, it is crucial to analyze the instability from charge traps since the long-term operation of the devices may be affected [12,13,14,15,16,17]. The bias stress effect is attributed to the continuous application of a gate voltage for a long period of time [18]. In general, this causes a shift of $V_{t}$. The bias stress instability either leads to a degradation in which the stress effect is reflected as a hysteresis, or a bias stress effect occurs irreversibly with the application of $V_{g}$.

The hysteresis observed in OFETs is the variation of the measured current, which changes the value of $V_{t}$ based on the sweep direction of $V_{g}$. Hysteresis can be evaluated as the difference between the two when the source-drain current $I_{d}$ causes forward and reverse sweeps of the gate voltage $V_{g}$. Hysteresis is not an unwanted feature, despite being undesirable for regular transistor operation, since it presents the potential for nonvolatile memory devices [19,20,21]. However, it is still one of the factors that deteriorates the OFET performance in several areas, and it is thus better to avoid it if possible. Therefore, in this study, we analyze the charge trap, which is the one of the most important causes of hysteresis, and we present a brief summary of the previous studies that have been conducted on the correlation between charge traps and hysteresis.

The main contribution of this paper are as follows:

(a) First, we perform a quantitative analysis of the hysteresis in OFETs. In particular, we generalize the current–voltage curve using one parameter that can be classified into linear and saturation regimes according to a new parameter to measure the hysteresis. We also introduce two measurement methods that quantitatively measure the degree of hysteresis in the form of a transfer curve. This enables the analysis of the causal relationship and correlation with charge traps in the future.
(b) Second, we summarize the factors affecting hysteresis in OFETs. We then focus on the charge trap as one of the major causes of hysteresis and analyze the various origins of the traps in OFETs to obtain a better understanding of the effect of the traps on hysteresis. Subsequently, we develop various methods to estimate the trap density of state (DoS) using trap models generated at the semiconductor/dielectric interface. The electron and hole traps, which directly affect hysteresis, are analyzed based on this estimation, and the dynamics of the change in the threshold voltage due to these traps, which causes hysteresis over time, are also determined.
(c) Lastly, we introduce various causal inference methods to quantitatively analyze the causal relationship between the traps and hysteresis as a data-based machine learning approach, which is difficult to address through only physical experiments. One effective approach involves determining the cause-and-effect relationship of each variable based on the data obtained through physical experiments. This method can be easily implemented to analyze the causal relationship between the two in the future since it can be estimated even for data that are not well observed.

The remainder of this paper is organized as follows. Section 2 presents a discussion on the measurement and quantification methods for hysteresis. Additionally, we summarize the causes of hysteresis in OFETs. Section 3 first describes the origins of charge traps and then presents a detailed description of the density estimation models for these charge traps. Section 4 presents a discussion of the effect of traps on hysteresis in static and dynamic cases over time. Section 5 introduces causal inference, a data-based machine learning analysis method, which is implemented for trap and hysteresis correlation and causal analysis. Section 6 concludes the study. We present a relationship analysis between hysteresis and charge traps. Furthermore, this focused review suggests that the effects of charge traps and quantitative analysis can be achieved by means of machine learning-based data analysis including casual structure analysis. 

## 2. Measuring and Quantifying Hysteresis

In this section, we briefly explain the hysteresis that occurs in OFETs and discuss the causes and measurement parameters of the hysteresis.

### 2.1. Transfer and Output Characteristics in OFETs

**Figure 2:**
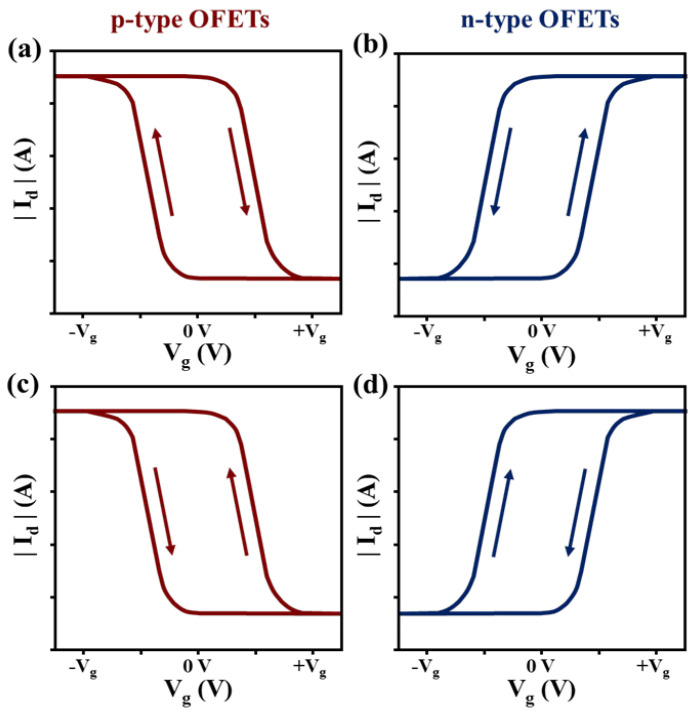
Example of transfer characteristics of p-type (**a**,**c**) and n-type (**b**,**d**) in OFETs. For each type, (**a**,**b**) depict higher back sweep current hysteresis whereas (**c**,**d**) show lower back sweep current hysteresis.

**Transfer Characteristics.** As explained in the previous section, hysteresis is considered as the dual sweep transfer characteristic of the drain current $I_{d}$ corresponding to the gate voltage $V_{g}$, where $I_{d}$ varies based on the sweep direction of $V_{g}$, as shown in Figure 2. This kind of bistability phenomenon is observed frequently in OFETs [22]. Depending on its subtle effect, hysteresis creates a backward sweep current that changes the sweep from an on state to an off state, which is smaller or larger than that of the forward sweep that changes the sweep from an off state to an on state.

Figure 2a,b depict the schematic transfer curves, from which we can observe the clockwise direction for p-type OFETs, whereas it is anticlockwise for n-type OFETs. In general, the latter case comes from the charge carrier trapping near the channel, whereas the former case typically results from the mobile ions or the polarization in the dielectric [19]. Figure 2c,d depict p-type and n-type transfer curves opposite from those presented above. Conversely, the hysteresis loop can also be caused by a shift in $V_{t}$ due to the bias stress [22,23]. Bias stress occurs if a constant gate voltage is applied for an extended period of time. This results in instabilities and hysteresis when there is a large extent reversible with $V_{g}$. If the bias stress effect is irreversible with $V_{g},$ it causes a deterioration [19]. Recovery following a power law corresponding to time occurs within a few days under dark conditions [23]. Several studies have been conducted to analyze the threshold voltage shifts. Most of them have suggested that the observed shift may be caused by the traps at the interface [24]. The hysteresis size (from the $V_{t}$ in the off-to-on sweep to the $V_{t}$ in the on-to-off sweep) may depend on the start and end voltages of the sweep, sweep rate, step width, delay/hold time, applied voltage sweep range, and measurement time [25,26]. However, it is very challenging to directly compare different devices when these various and complex parameters are not well defined.

**Output Characteristics**. Contrary to the transfer characteristic ($I_{d}$,$V_{g}$), which is a curve of $I_{d}$ corresponding to the gate voltage $V_{g}$, the output characteristic is a pair of ($I_{d}$,$V_{d}$), that is, drain current $I_{d}$, corresponding to the drain voltage $V_{d}$. These two characteristics typically exhibit the following relationships:

(1) $$I_{d}=\frac{W}{\alpha L}\mu_{0}C_{i}{\left(V_{g}-V_{t}\right)}^{\alpha }V_{d}^{2-\alpha }.$$

**Figure 3:**
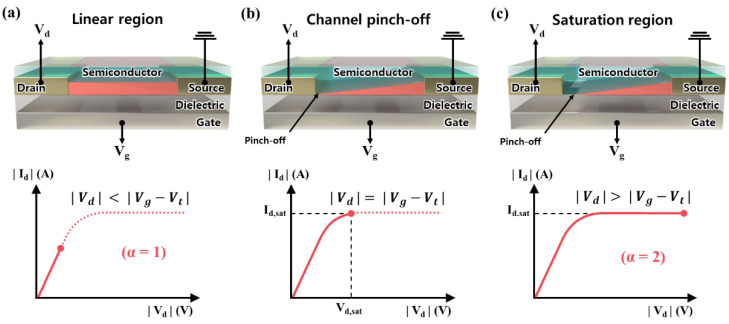
The α-regime in OFET corresponding to ($I_{d}$, $V_{d}$ ) characteristics. ((**a**) Linear regime (α=1), (**b**) Channel pinch-off, and (**c**) Saturation regime (α=2)).

Here, $\alpha \in \left\{1,2\right\}$ represents a parameter that separates the linear regime ($\alpha =1\;for\;\left|V_{d}\right|<\left|V_{g}-V_{t}\right|)$ and the saturation regime ($\alpha =2\;for\;\left|V_{d}\right|\geq \left|V_{g}-V_{t}\right|)$. Therefore, in this paper, we refer to this as the “α regime”. W and L represent the channel width and length, respectively, as shown in Figure 1, and $\mu_{0}$ represents the field-effect mobility. Here, $C_{i}$ represents the capacitance per unit area of the dielectric. In the α regime, for α=1, we observe that the drain current $I_{d}$ increases linearly with the increase in $V_{d}$ since $V_{d}^{2-\alpha }=V_{d}^{2-1}=V_{d}$ in Equation (1), which is also depicted in Figure 3a. For α=2, the drain voltage becomes constant, that is, $V_{d}^{2-\alpha }=V_{d}^{2-2}=V_{d}^{0}=1$, so the drain current does not increase regardless of any value of $V_{d}$, as shown in Figure 3c. For the case of $\left|V_{d}\right|=\left|V_{g}-V_{t}\right|$, the condition is referred to as “pinch-off,” as shown in Figure 3b. 

In Figure 3a, for α=1 ($\left|V_{d}\right|<\left|V_{g}-V_{t}\right|)$, we see the linear regime of the device. This means that $I_{d}$ linearly increases as a function of the drain-source voltage. If $V_{d}$ increases and the magnitude approaches the magnitude of $\left|V_{g}-V_{t}\right|$, the two potentials enable the modulation of the channel conductivity, and this change is based on the magnitude of the threshold voltage [4]. When $V_{d}$ reaches the saturation point $V_{d,sat}$, which is the point that satisfies $\left|V_{d}\right|=\left|V_{g}-V_{t}\right|$, free charge carriers disappear in the region near the drain side, and accordingly, the pinched-off phenomenon occurs as shown in Figure 3b. This results in an electric field to the drain electrode at the pinch-off point, which causes a space charge limiting current. Lastly, as $V_{d}$ increases, it creates a competing effect of increasing the potential, forcing charge from the source to the drain, and a larger depletion region near the drain causes $I_{d}$ to saturate, as shown in Figure 3c; the saturation regime is α=2 ($\left|V_{d}\right|>\left|V_{g}-V_{t}\right|)$ [4].

### 2.2. Hysteresis Parameters of Measurements

Hysteresis is based on the difference between the forward and backward sweeps in the ($I_{d}$, $V_{g}$) transfer characteristics, as explained earlier. However, it is more important to establish a quantitative indicator of the amount of hysteresis, rather than simply understanding the degree of hysteresis based on the visual interpretation of the graph. In this subsection, we introduce two representative quantitative measurement methods of hysteresis: (1) hysteresis index [27,28] and (2) slope difference [29]. 

**Hysteresis Index:** Hysteresis not only occurs in semiconductors, such as OFETs, but it is also a common phenomenon in various fields that handle electrical properties, such as solar cells [27,28]. Consequently, we have analyzed the numerical induction of hysteresis corresponding to the sweep phenomenon [27,28]. This method is called the hysteresis index (HI), which is calculated as the difference in the $I_{d}$ value based on the change in voltage for both the forward and backward sweeps in the transfer curve. 

HI is expressed as follows:

(2) $$\mathrm{Hysteresis}\;\mathrm{Index}\;(\mathrm{HI})=\;\frac{\int_{t}^{s}|(I_{d}^{f}\left(V_{g}\right)-I_{d}^{b}\left(V_{g}\right)|dV_{g}}{\int_{t}^{s}I_{d}^{max}\left(V_{g}\right)dV_{g}}$$

**Figure 4:**
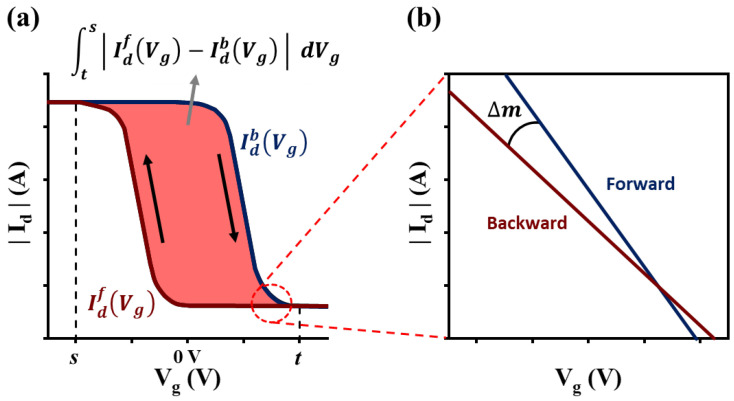
Hysteresis metrics: (**a**) hysteresis index [28] and (**b**) slope difference [29].

In Equation (2), $I_{d}^{f}\left(V_{g}\right)$ and $I_{d}^{b}\left(V_{g}\right)$ represent the drain currents in the forward and backward directions, respectively, as shown in Figure 4a. In the integral of the equation, t denotes the threshold voltage point in the backward direction and s denotes the sweep point. Here, $I_{d}^{max}\left(V_{g}\right)=\max\left\{I_{d}^{f}\left(V_{g}\right),\;I_{d}^{b}\left(V_{g}\right)\right\},$ which is the maximum of the forward and backward currents. Consequently, the value of HI is [0, 1]. Thus, HI is defined as the ratio of the difference between the two values to the sum of the large current values in t and s. A higher HI value indicates a more serious hysteresis.

**Slope Difference:** The slope difference Δm as a hysteresis parameter was introduced in [29], which presents information based on the charge trapping behavior. This parameter can be obtained by means of the slope of the measured transfer curves as shown in Figure 4b. To present a more detailed description of this concept, we consider the gate voltage range as $\left[-V_{max},\;V_{max}\right]$, which indicates that the forward sweep goes from $V_{g}=0$ to $V_{g}=-V_{max},$ and the backward sweep goes from $V_{g}=-V_{max}$ to $V_{g}=0$. Hence, a gate-voltage sweep occurs at $V_{g}=-V_{max}$. Let ${\left(dI_{d}/dV_{g}\right)}_{-V_{max}^{-}}$ be the derivative of the transfer curve, $I_{d}$, to the sweep point, $V_{g}=-V_{max}$, by varying $V_{g}$ to the left, and let ${\left(dI_{d}/dV_{g}\right)}_{-V_{max}^{+}}$ be the derivative at the same point but in the opposite direction of the transfer curve. Then, the slope difference Δm is computed as

$$\Delta m={\left(\frac{dI_{d}}{dV_{g}}\right)}_{-V_{max}^{-}}-{\left(\frac{dI_{d}}{dV_{g}}\right)}_{-V_{max}^{+}}=2k\frac{V_{max}^{\beta }}{a^{\beta }\tau^{\beta }}\frac{I_{d}\left(-V_{max}\right)}{V_{max}}$$

where a denotes the scan rate, β (0<β≤1) denotes the dispersion parameter, and τ denotes the trapping-time constant. The constant k denotes a parameter that scales corresponding to the amount of the trap density. Therefore, the corresponding metric will have a larger slope value at the turning point for severe charge trapping. 

The degree of hysteresis can be expressed with one piece of numerical information since the two hysteresis parameters presented above correspond to real values. The correlation and causality between the hysteresis and charge traps can be quantitatively measured, as will be explained in the later sections of this paper.

### 2.3. Causes of Hysteresis

In this subsection, we discuss the causes of hysteresis. In previous studies, it was observed that hysteresis in OFETs is caused by various factors. Among these, charge trapping or detrapping near the interface of the semiconductor and dielectric is the most significant factor [14].

**Figure 5:**
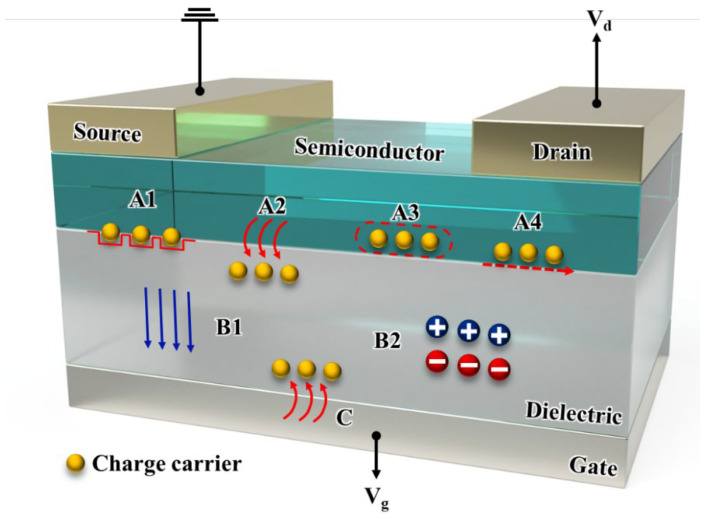
Scheme of mechanisms that cause hysteresis with BGTC structure.

The authors of [19] analyzed the causes of hysteresis from various perspectives. They primarily focused on the bottom gate and classified the causes into several categories, such as traps, bulk effects of the dielectric, and charge injection, which are depicted in Figure 5, as follows:

1. **Semiconductor/dielectric interface traps** (A1): Several traps occur at the semiconductor/dielectric interface in OFETs. These traps are caused by various factors such as impurities, structural defects, and self-trapping [19]. An example of a structural defect, the effective conjugation length of a polymer, can lead to some change of energy levels [19,30,31,32]. Additionally, when the rate of charge release in these traps is low enough, the sweep rate exceeds the time required to increase the thermal equilibrium, resulting in a hysteresis on the device [32].
2. **Charge injection from the semiconductor channel into the dielectric** (A2): In OFETs, it is observed that charge can be injected from the semiconductor into the dielectric. Although this type of injection is not a charge trap, from a device perspective, it still works as a trap that produces hysteresis [19]. For example, in floating-gate transistors, the injected charge is stored in the floating metal layer semipermanently, and it affects the gate field. This may lead to a change in $V_{g}$ of the transistor, which produces hysteresis [33].
3. **Slow reactions of mobile charge carriers** (A3) **and mobile ions in semiconductors** (A4): There is a decrease in the sweep speed (measuring slower), which increases the hysteresis, indicating a lower mobility, as shown in Figure 3. Furthermore, the mobile ions can be considered as the fourth reason for the hysteresis (A4) [14]. This is contrary to hysteresis generated by mobile ions in the dielectric. Mobile ions in the semiconductor move slowly toward the channel with the same polarity as the majority carrier. Reducing the number of mobile charges by changing the number of ions in the channel decreases $I_{d}$, which causes hysteresis [34].
4. **Polarization of the dielectric** (B1) **and mobile ions in the dielectric** (B2): In the case of an externally applied electric field, ferroelectric dielectrics exhibit remanent polarization, which generates an electric field along with the gate field; thus, ferroelectric dielectrics also cause hysteresis [14]. Furthermore, the mobile ions in the dielectric on the device give similar effects to those observed for the polarization of the dielectric. The hysteresis caused by this can be clearly observed for the OFET.
5. **Charge injection from the gate** (C): Hysteresis is also caused by the charge injection between the gate electrode and the dielectric. In [19], the authors demonstrated that electrons were injected in the on state (negative $V_{g}$). When $V_{g}$ is reduced to 0 V, the electrons remain in the dielectric and stabilize the accumulated holes, which form the channel. A large hysteresis can be produced by electrons remaining for only a short period along with a fast sweep rate of the electrons [35]. Conversely, the rate of slower sweep decreases the hysteresis. It has been observed that floating gate transistors mainly use charge injection as the dielectric of the semiconductor [21,33].

In the following Table 1, we categorize the three typical mechanisms that cause hysteresis as well as their sources.

Thus, we demonstrate that although hysteresis is caused by various factors, the charge trap is a very important factor. In the next section, we introduce the causes of these charge traps in organic semiconductors and explain the analysis of trap density.

## 3. Charge Traps and Analysis

### 3.1. Charge Traps and Their Origins

**Figure 6:**
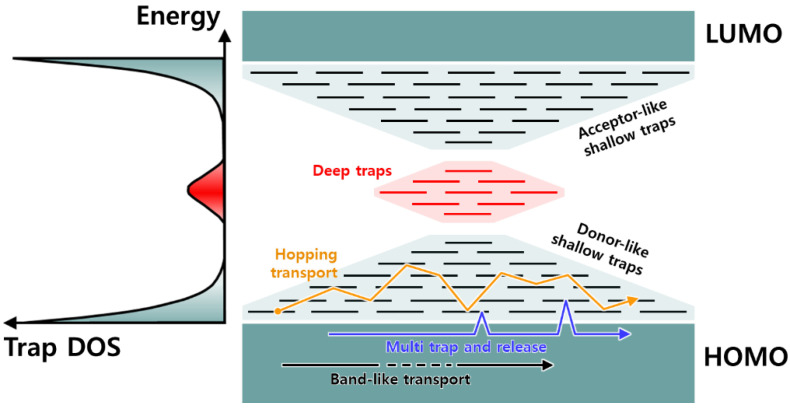
Schematic diagram of trap DoS function and corresponding trap states (shallow trap and deep trap).

**Charge Trapping:** Extensive research has been conducted for several years on the charge traps in organic semiconductors since the traps that inevitably occur during manufacturing and processing affect the performance of OFETs [23,31,32]. A charge trap is a type of defect in OFETs that generates local electronic states that are spatially distributed around the defects and energetically distributed in the semiconductor [32]. Therefore, the trap density of state (DoS) function, which is a kind of energy distribution for the electronic state, should be clearly discussed to get a better understanding of the charge trap. The DoS can be approximated by a Gaussian or exponential distributions in disordered semiconductors [36,37]. For a Gaussian DoS, the transfer of effective energy is the energy generated when charge carriers hop multiple times between local states over time [38,39]. In electron energy state analysis, there are two similarities, known, respectively, as the top of the valence band and the bottom of the conduction band for the highest occupied molecular orbital (HOMO) and lowest unoccupied molecular orbital (LUMO) [32]. The HOMO and LUMO can be differentiated by using the trap DoS and energy state graphs, as shown in Figure 6. The charge traps can be divided into two different patterns based on these energy states: (1) **shallow traps** and (2) **deep traps**. This classification is dependent on the relative energy position from the trap depth at a specific temperature [32]. Shallow traps occur near the edge of the band, whereas deep traps are observed farther from the edge of the band. In Figure 6, the trap DoS function indicates the two types of traps: the first one is a shallow trap (black), which is located at the tail states of the energy, and the second one is a deep trap (red) in the bandgap. It is observed that the tail states seem acceptor-like and donor-like. However, most of the studies conducted on traps have been primarily focused on shallow traps since deep traps are very difficult to observe and infer.

In Figure 6, we see that there are three types of transport mechanisms: (i) band-like transport, represented by a black arrow; (ii) multiple-trap and release (MTR), represented by a blue arrow; and (iii) activated hopping transport between localized states thermally, represented by an orange arrow [32]. Traps temporarily hold charge carriers until they are ejected back into the band due to an external excitation, such as an electric field or a photon. In particular, charges traveling within the delocalized state can be trapped by shallow traps, which is a localized state in the bandgap, and then return to the energy band in the MTR model [40,41]. As the DoS increases, although charge carriers are confined, they can participate in transport through active hopping or tunneling, as indicated by the orange arrows in Figure 6 [42]. Next, we analyze the various causes of these traps.

**Trapping Sources:** Traps in OFETs are produced by various sources, as shown in Table 2. Typically, trap sources are classified into intrinsic or extrinsic sources. In terms of an intrinsic origin, the most significant source of traps in OFETs is disorder [32]. The disorder originates from the local destruction of the crystal structure due to perturbations or imperfections of a single or several unit cells, which causes the destruction of long-range order. For example, there is a disorder called off-diagonal disorder, which is caused by the structural property of bonding electrons between molecules [43]. A structural disorder in the Cartesian domain inevitably causes a trap DoS, which causes the charge trapping sites in its energy domain [39]. Typically, energetic disorder can be modeled by using a Gaussian distribution [44]. Furthermore, the exponential trap DoS is also used to model the tail states in the band gap [36,45]. There are two types of disorder: (i) static disorder and (ii) dynamic disorder. Static disorder comes from some structural defects and chemical impurities, whereas dynamic disorder occurs due to changes over time. Both types of disorder are considered intrinsic sources of traps [32]. The primary difference between the static and dynamic disorder is that dynamic disorder produces time-dependent changes in energy and occurs across the entire crystal, whereas static disorder is observed only at specific locations regardless of the time. The position of the carrier in dynamic disturbance can be found if the electronic energy is destroyed [46]. Dopants are another intrinsic source of energy traps, and they are used in OFETs to improve the conductivity. The charge transport in doped OFETs is very complex and is achieved by various methods. For example, adding a dopant can extend the host trap DoS to introduce a tail state, a dopant-induced charge transportation can fill and neutralize an existing trap state, or the existence of a dopant can cause the extinction of a trap state [47,48].

Extrinsic traps result from gases, external light, applied voltage, or a combination of other materials [32]. Among these, the interface trap that occurs at the semiconductor/dielectric interface is one of the most important factors. In OFETs, the formation of a transistor channel and charge traps can degrade the performance of these devices if present in the gate dielectric or at the interface between the semiconductor and the dielectric [4]. For example, some nonuniform morphologies scatter the accumulated charges [49,50], resulting in structural defects produced by the surface energy and chemical properties along with the dielectric layer roughness or morphological arrangement of the semiconductor deposited on top. Traps are also created at the semiconductor/dielectric interface by impurities from adsorption of water, oxygen, etc. A dangling bond can be formed on the surface of the SiO_2_ gate dielectric surface caused by adsorption of hydroxyl groups, resulting in the capture of electrons [32]. This has been the main challenge limiting the realization of electron transport in SiO_2_-based transistors [51]. When device fabrication is in progress, especially when exposed to the external environment, the exposure can often affect the quality of organic semiconductors and cause trap formation. The trap caused by temperature in OFETs leads to shifts of turn-on voltage $V_{on}$ subthreshold slope S, and threshold voltage $V_{t}$ [52,53]. The ambient moisture in pentacene films can cause OFET device degradation, producing larger $V_{t}$, S, and high on-currents [54,55,56,57]. Refer to [32] for a detailed explanation and analysis of each of the causes of the intrinsic and extrinsic trapping sources. 

### 3.2. Trap DoS Analysis

The trap DoS must be investigated to obtain a better understanding of traps in OFETs. However, it is difficult to characterize the trap DoS, regardless of how well the OFETs estimate the origin of traps. Consequently, various device manufacturing and measurement technologies have been developed to obtain accurate experimental data, making it possible to estimate the trap DoS. However, the results are not always consistent as these technologies cover cases where the approximation can vary and the energy distribution is not uniform [32]. In this subsection, we summarize the estimation approaches as follows: (1) **interface trap density estimation** and (2) **other trap density estimations**. 

**Figure 7:**
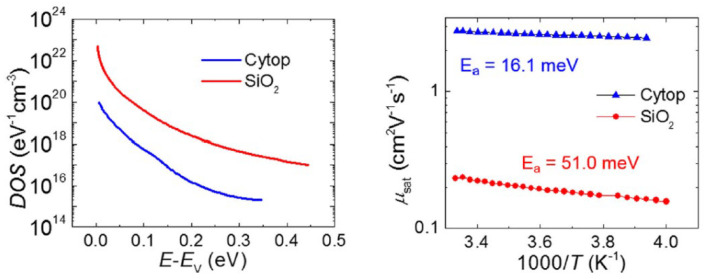
(**Left**) Trap DoS in diF-TES ADT OFETs with Cytop and SiO_2_ dielectrics. (**Right**) Arrhenius plots for both Cytop and SiO_2_. Reproduced with permission [59]. Copyright © 2015, AIP Publishing.

**Interface Trap Density Estimation:** As explained earlier, the discontinuous contact structure of the OFETs between the semiconductor and dielectric causes traps to inevitably occur at this interface, which significantly affect the performance. The trap DoS at the semiconductor/gate dielectric interface must be characterized to obtain specific operating principles and insight into how charge is transported in organic semiconductors [58]. Consequently, the authors in [58] quantitatively determined the interface trap density of a rubrene single-crystal field-effect transistor with two kinds of interfaces by using the gate bias stress technique. In their work, the reversible and reproducible current-voltage (I-V) characteristic shifts characterized by changing traps and detraps at the crystal/SiO_2_ interface were observed under both the negative and positive gate bias stresses. Thus, they can obtain a quantified result of trap DoS at the interface that alternately fill and empty with relative accuracy within a period of approximately one hour over a range of energies defined by the applied bias stress. The authors of [59] obtained the electrical properties of OFETs with SiO_2_ and fluoropolymers (Cytop) as top and bottom gate dielectrics. They used 2,8-difluoro-5,11-bis(triethylsilylethynyl)anthradithiophene(diF-TES ADT) as a semiconductor material to fabricate transistors considering diF-TES ADT/Cytop, diF-TES ADT/SiO_2_ interfaces. They used Grunewald’s approach to get the trap DoS [60,61] and obtained the experimental results for the trap DoS with the Cytop and SiO_2_ dielectrics, respectively, as shown in Figure 7. In the left figure, we can observe a rapid decrease in the trap DoS in the band gap in the Cytop-based devices. The right figure depicts the Arrhenius plot, which is obtained for the activation energy, with 51.0 meV for the SiO_2_ OFETs, and 16.1 meV for the Cytop top gate OFET. They observed that Cytop is chemically inert due to its fluorinated nature, and it is highly hydrophobic, which helps in reducing the contaminants that can alleviate the trap DoS at the semiconductor/dielectric interface.

Subsequently, we discuss how the interface trap DoS is calculated at the semiconductor/dielectric interface. We first consider a subthreshold swing, which is a device parameter to quantify how rapidly a transistor can transition from an off state to an on state by a gate voltage $V_{g}$. In particular, the subthreshold swing S is defined as the change (derivative) in the gate voltage corresponding to the induced drain current change by $S=dV_{g}/d\left(logI_{d}\right)$ [62]. Furthermore, we assume that both the density of the deep bulk trap, $N_{bulk}$, and the interface trap, $N_{it}$, are independent of energy [63]. If the bulk trap is ignored, after basic calculus, we obtain the maximum interface trap density $N_{it}$ as follows:

(3) $$\;N_{it}=\left[\frac{Slog\left(e\right)\;}{kT/q}-1\right]\frac{C_{i}}{q}$$

where k denotes the Boltzmann constant, q denotes the elementary charge, T denotes the absolute temperature, and $C_{i}$ denotes the dielectric capacitance per unit area. The authors [62] obtained the trap density at the interface using devices based on this formula.

**Other Trap Density Estimations:** In addition to the interface trap DoS, several analytical methods have been proposed to obtain the trap DoS from the transfer characteristics of OFETs [64]. The authors in [64] conducted a study to estimate the trap DoS from various perspectives They first applied the most widely used methods to the transfer characteristics. They then implemented a computer simulation program to compute the trap DoS, which produces simulated transfer characteristics that approximate the measured data. For this purpose, they considered pentacene and p-type conduction. The trap DoS calculation method can be applied in the case of charge transport, which characterizes the distribution of local states below a certain level [65,66]. Furthermore, if a more sophisticated description is used, the basic trap DoS can be quantified by using a FET. In the survey [65], the linear-regime transfer characteristics were considered for several analytical methods, and some assumptions were made for mathematical tractability.

1. The charge density across the transistor channel from source to drain is uniform.
2. The Fermi function for the trapped hole uses a zero-temperature approximation step function.
3. The valence band edge with the occupancy and effective density of the extended state using the Boltzmann function is approximated as a discrete energy level.
4. The dependence on temperature of the Fermi energy and interface potential was neglected (neglecting the statistical shift).

Here, especially for the first assumption, the case of a drain voltage having poor transfer characteristics is considered. In this case, an “unperturbed” condition can be assumed in a metal-insulator-semiconductor (MIS) structure, but there is no drain voltage applied [64]. Table 3 presents the summary of the different methods. (In the table, $N\left(E\right)$ indicates the trap DoS with respect to the energy.)

**Figure 8:**
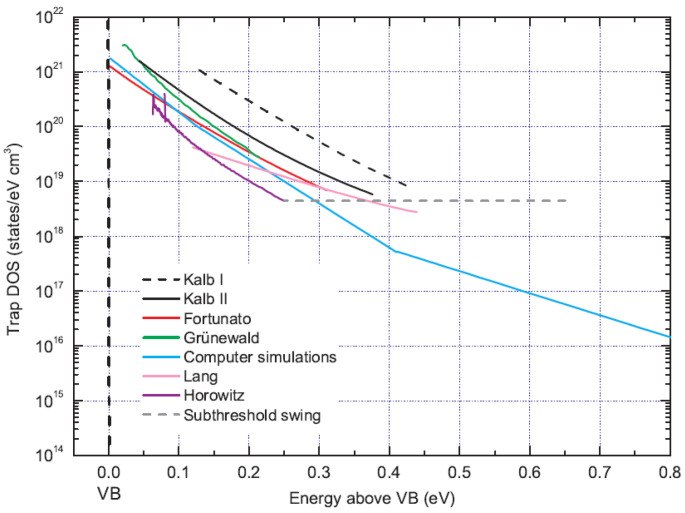
Comparison of the interfacial trap DoS obtained for pentacene thin-film transistors using several analytical and numerical methods. Reproduced with permission [64]. Copyright © 2010, American Physical Society.

Figure 8 depicts the trap DoS, which is calculated by using different methods on the same measurement dataset. In the result, the relative energies of the valence band edges are taken into account (VB). In the figure, estimates smaller than S are assumed to be independent of trap DoS and energy, especially for traps that are only slightly higher than the Fermi energy. Furthermore, it is verified that the trap DoS increases exponentially with energy. This demonstrates that the initially selected model and parameters affect the final results. Therefore, it is crucial to select an appropriate method to calculate the trap DoS [64].

However, these results assume that the trap DoS at S is energy independent, and they provide only rough estimates for traps near some quasi-Fermi levels. Further, the accuracy of all curves depends on the complete Fermi-Dirac statistics used in the simulations. These results demonstrate that the trap density is estimated to be smaller than it actually is because in the method proposed by Lang et al., the dependence of the layer thickness on the gate voltage, evidenced by the slope of the curve near the band edge, is neglected. Furthermore, the methods corresponding to the temperature dependence of band mobility proposed by Kalb et al. and Fortunato et al. presented similar simulation results [64].

## 4. Hysteresis from Traps

In this section, we analyze the two types of traps that cause hysteresis (electron and hole traps) and the reason behind the occurrence of hysteresis. We then present the simulation results for hysteresis caused by the charge trap. Lastly, we discuss the dynamic analysis of hysteresis corresponding to the trap characteristics that change over time.

### 4.1. Electron Traps

**Figure 9:**
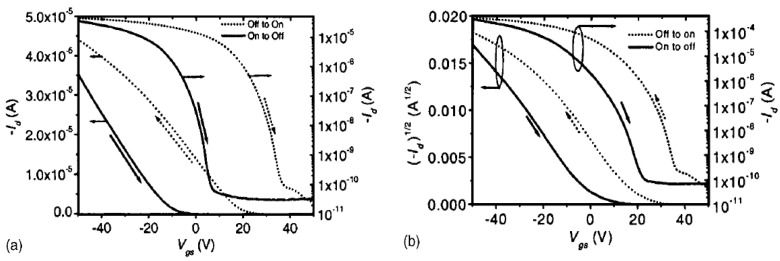
Transfer characteristics with a device L=50 μm and W=1500 μm for (**a**) the linear regime ($V_{d}=-10\;V)\;$ and (**b**) the saturation regime ($V_{d}=-50\;V)$, respectively. Reproduced with permission [73]. Copyright © 2005, AIP Publishing.

The electron traps in pentacene are considered long-lifetime minority traps that fill quickly and empty slowly. These electron traps cause hysteresis [19]. Some studies have focused on the deep electron traps for pentacene on SiO_2_ [72,73]. In those studies, all the electron traps were empty when the sweep started in the “on” state (in this case, $V_{g}$ is negative for pentacene). Next, if an off-voltage is applied, (positive $V_{g}$ is applied), it quickly fills the trap. Subsequently, due to rapid sweeping, a negatively charged trap generates a more positive charge than the one corresponding to the given $V_{g}$ field. The overflow of holes produces a higher $I_{d}$ in the forward sweep. While in the “on” state, all the traps were emptied, reducing $I_{d}$ on the backward sweep. Faster forward sweeps result in a higher number of filled traps with larger $I_{d}$, which explains the increased magnitude of the hysteresis for faster sweeps. In [73], the authors assumed that negative charges stored in semiconductors, possibly trapped electrons, are responsible for the memory effect. This proposition was proved by using time domain measurements, which were performed by using a high-quality SiO_2_ gate dielectric to avoid gate dielectric effects. In Figure 9, it can be observed that the transfer curve is swept between $V_{g}=-50\;V$ and $V_{g}=50\;V$ in steps of 1 V. It is demonstrated that this loop-wise hysteresis was always observed for OFETs when using a thermal SiO_2_ dielectric.

The authors of [73] made the following conclusions:

- The results were dominated by a long-lifetime deep electronic support at the pentacene or interface. The negative charges initially accumulated in the channel during the off-on sweeps, and then the traps were filled. During $V_{g}$ sweeps to negative bias, hole carriers were generated more than currently required for $V_{g}$ and the gate channel capacitance. Since the net charge satisfied the charge-voltage relationship, there must be an additional hole to balance the negative charge. Conversely, with hole accumulation, the on-to-off sweep began; hence, there was no stored negative charge. Thus, additional holes generated additional $I_{d}$ during the off-on sweep for the same $V_{g}$.

### 4.2. Hole Traps

**Figure 10:**
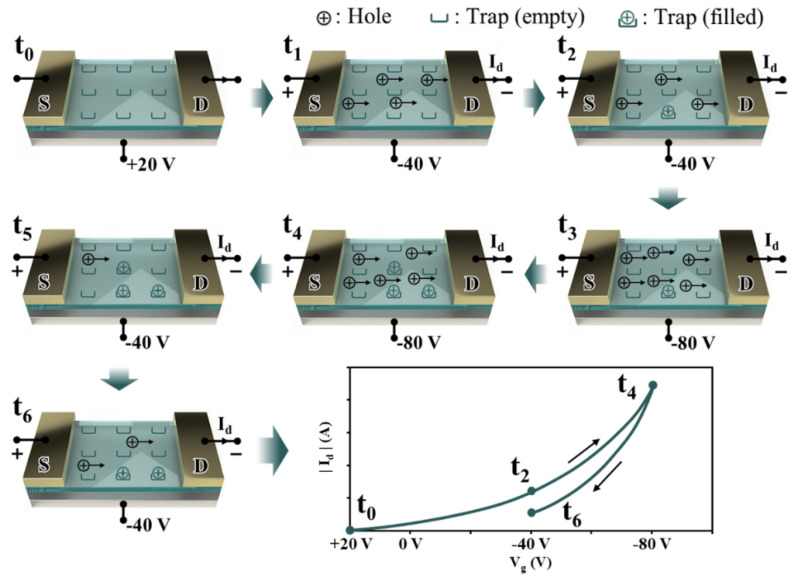
Pentacene FET transient behavior during a gate-voltage sweep [74].

Pentacene hole traps can cause low hysteresis, with most traps filling quickly and emptying slowly [19]. In [74], the authors introduced a model that correlated with the hysteresis of the pentacene transistor affecting the active layer hole trap. They analyzed the hysteresis mechanism in the bottom gate, bottom contact (BGBC) pentacene OFETs. In their study, a mixed configuration with SiO_2_ comprising the gate dielectric was used to avoid the effect of gate insulation. They observed that the traps were empty when the sweep started from the off state. However, the traps started to fill within the off-to-on sweep. For pentacene OFETs, in the case of $V_{g}$ with a negative value, this corresponds to electric-field-induced holes, with some being trapped at high speeds. At given $V_{g}$ and $I_{d},$ some mobile holes in the channel decreased since the trapped holes were released slowly during on-to-off sweeps (much slower than the sweep speed) [72,73]. The trap release rate must be lower than the sweep rate. Essentially, fast sweeps present larger hysteresis than that of slow sweeps. This sweep rate is considered a key property between the different hysteresis mechanisms [74]. Figure 10 shows the behavior of the charge distribution during a gate-voltage sweep in a pentacene FET from 20 V to −80 V and back to −40 V. During this process, $V_{d}$ was set to a constant negative value. The following section describes the change in the current when the gate voltage changes at each time interval.

In Figure 10, we observe the positive gate voltage $V_{g}$ without accumulated holes; essentially, the hole traps are empty at $t=t_{0}$. If the gate voltage is changed to a negative voltage—that is, $V_{g}$= −40 V, with $\left|V_{g}|>|V_{t}\right|$—the accumulation layer at the dielectric-semiconductor interface creates a conductive channel, through which the hole current flows ($t=t_{1})$. However, parts of the accumulated holes become trapped over time, resulting in a usable free-charge carrier density. Consequently, the current $I_{d}$ is reduced $\left(t=t_{2}\right)$. When a more negative gate voltage is applied—that is, $V_{g}$= −80 V—the hole density and current $I_{d}$ begin to increase ($t=t_{3})$, but then they decrease again as more holes are trapped $\left(t=t_{4}\right)$. Subsequently, when $V_{g}$ switches to $V_{g}=$ −40 V, the current $I_{d}$ begins to decrease ($t=t_{5})$ and then increase with time due to the released trapped holes. At $t=t_{6}$, the flow of the current is less when compared to that at $t=t_{2}$, which comes from the larger number of trapped holes generated by the sweep at $V_{g}$= −80 V. Consequently, the $I_{d}$ of a pentacene FET depends on both the bias conditions and on the bias property of the device owing to hole trapping in the semiconductor.

### 4.3. Simulated Effects from Trap Charging

**Figure 11:**
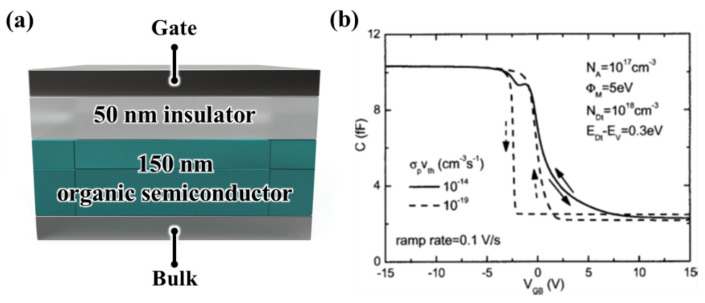
(**a**) Simulated MOS structure and (**b**) simulated CV characteristics for acceptor doping and donor-like trap states near the valence band edge. Reproduced with permission [75]. Copyright © 2005, AIP Publishing.

The authors in [75] analyzed hysteresis in OFET devices as a simulated effect of trap charging. They conducted this study to clarify one of the proposed mechanisms, i.e., the field effect hysteresis, that can occur due to trap recharging through numerical two-dimensional (2D) simulations. The simulation proposed by the authors was first performed for a metal oxide semiconductor (MOS) capacitor, as shown in Figure 11a. Figure 11b depicts the resultant capacitance-voltage (CV) characteristics. The authors calculated and analyzed the quasistatic CV characteristics. They first assumed the existence of traps with different distributions and adjusted the parameters in the extensive simulations of the quasistatic CV characteristics in MOS capacitors with organic semiconductors, which were performed by using the drift-diffusion model. For this purpose, ISE-TCAD, a two-dimensional device simulation program, was used. The authors considered a field-effect device in their simulations and made the program simultaneously solve Poisson’s equation for the electric potential. For the hole and electron densities, they used the continuity. Particularly, trap states such as an additional ionization acceptor-like or donor-like state in the vicinity of the valence or conduction band, respectively, or a discrete donor or acceptor without basic doping were considered first, corresponding to the parameters for the trap state. Additionally, they assumed that (1) there simultaneously exist an acceptor-like dopant near the valence band edge and an individual acceptor-like trap state, and (2) there exists a donor-like trap near the conduction band edge along with a donor-like dopant.

In the simulated model, the authors considered donor-like traps at an energy 0.3 eV above the valence band edge and a concentration of $10^{18}{\mathrm{cm}}^{-3}$, which is larger than the doping concentration of $10^{17}{\mathrm{cm}}^{-3}$ of completely ionized acceptors [76]. Bulk neutrality is provided by positively charged traps in which acceptors exist with a Fermi energy 0.355 eV higher than the valence band and a hole concentration approximately two orders of magnitude lower, $10^{15}{\mathrm{cm}}^{-3}$ [75].

The results presented in the figure demonstrate that hysteresis is not observed for the standard value of $\sigma_{p}v_{th}=10^{-14}{\mathrm{cm}}^{3}s^{-1},$ where $\sigma_{p}$ denotes the capture of the cross section for holes and $v_{th}$ denotes the thermal velocity. However, the results in Figure 11b show that a small minimum appears after the peak, indicating that all traps are positively charged. Additionally, a final increase in the capacitance of the oxide results in additional hole accumulation. Subsequently, the shifted flat band voltage comes from band bending recharge, and charge traps that occur near the interface are illustrated. However, hysteresis is observed for an arbitrarily selected small value of $\sigma_{p}v_{th}=10^{-19}{\mathrm{cm}}^{3}s^{-1}$, as shown in Figure 11b, with a less negative flat band voltage for the sweep until it is depleted. Consequently, we conclude that the transition is relatively steep for both the sweep directions, indicating the concentration of a low mobile hole [75].

### 4.4. Dynamic Analysis of Hysteresis from the Traps

There have been many studies on the effect of traps on hysteresis for static cases. However, the static approach of hysteresis analysis is limited since the degree of trapping can change over time. To overcome this limitation, the authors in [29] first considered the time-varying condition of the charge trap phenomenon in OFETs and addressed it based on the following two approaches.

**Threshold Voltage Shift:** The authors proposed a dynamic method to quantify the number of traps in OFETs and the mechanism behind their formation. They analyzed the bias stress at the point where the transfer curve changes direction. More precisely, the bias stress is analyzed for the shift of $V_{t}$ from its starting value, $V_{t}\left(0\right)$, when $V_{g}\left(t\right)$ is constantly applied for the time duration t [77]. The changes are observed to follow an expansion exponential evolution over time, which is depicted as follows [76]:

$$\Delta V_{t}\left(t\right)=\left(V_{g}\left(t\right)-V_{t}\left(0\right)\right)\left[1-exp\left[-{\left(\frac{t}{\tau }\right)}^{\beta }\right]\right]$$

where *β* (0 < *β* ≤ 1) denotes the dispersion parameter and τ denotes the trapping time constant. Here, $\tau =\nu^{-1}exp\left(E_{A}/k_{B}T\right),$ where $E_{A}$ denotes the mean activation energy for trapping and ν denotes a frequency factor. Let $N_{0}\left(t\right)$, $N_{f}\left(t\right),$ and $N_{tr}\left(t\right)$ represent the surface charge densities, free carriers, and the number of trapped charges at time t, respectively. The conservation of the total amount of charges is

$$N_{0}\left(t\right)=N_{f}\left(t\right)+N_{tr}\left(t\right)=\left(V_{g}\left(t\right)-V_{t}\left(0\right)\right)C/q$$

Here, C denotes the capacitance of the gate dielectric, and q denotes the elementary charge. Subsequently, we obtain $\Delta V_{t}\left(t\right)=qN_{tr}\left(t\right)/C,$ which implies that if the trap DoS $N_{tr}\left(t\right)$ increases, the difference in the threshold value, $\Delta V_{t}\left(t\right)$, also increases. 

**Dynamic Behavior of Transfer Curve:** To observe the dynamic behavior of the transfer curve $I_{d}\left(t\right)$, the authors of [29] considered the linear regime ($\alpha =1\;for\;\left|V_{d}\right|<\left|V_{g}-V_{t}\right|)$ as follows:

$$I_{d}\left(t\right)=\frac{W}{L}\mu_{0}C_{i}\left(V_{g}\left(t\right)-V_{t}\left(t\right)\right)V_{d}$$

Here, the gate voltage $V_{g}\left(t\right)$ and threshold voltage $V_{t}\left(t\right)$ are time-dependent variables. Using the derivative corresponding to t, the authors obtained the following differential equation:

$$\frac{\partial I_{d}\left(t\right)}{\partial t}=\frac{W}{L}\mu_{0}C_{i}V_{d}\frac{\partial V_{g}\left(t\right)}{\partial t}-k\frac{t^{\beta -1}}{\tau^{\beta }}I_{d}\left(t\right)$$

where the second term originates from $d\Delta V_{t}\left(t\right)/dt\propto dN_{tr}\left(t\right)/dt\propto N_{f}\left(t\right)t^{\beta -1\;}/\tau^{\beta }$. For *β* = 1, the solution to the differential equation is as follows:

$$I_{d}\left(t\right)=I_{d}\left(0\right)\exp\left[-\left(\frac{k}{\tau }\right)t\right]+\frac{W}{L}\mu_{0}C_{i}V_{d}\frac{a\tau }{k}\left[1-\exp\left[-\left(\frac{k}{\tau }\right)t\right]\right]$$

Here, the trapping mechanism included the decay rate and the pre-exponential factor modulated by the gate-voltage sweeping rate a, $I_{d}\left(0\right)=-\frac{W}{L}\mu_{0}C_{i}V_{d}V_{t}\left(0\right)$; the short-time limit solution with $\tau^{\prime }:=\tau /k$ is given by

$$I_{d}\left(t\right)=\frac{W}{L}\mu_{0}C_{i}V_{d}\left(-at-V_{t}\left(0\right)\right)\exp\left[-\left(\frac{t}{\tau \prime }\right)\right]+\frac{W}{L}\mu_{0}C_{i}V_{d}\frac{a\tau }{k}\left[1-\exp\left[-\left(\frac{t}{\tau \prime }\right)\right]\right]$$

By taking the limit with respect to t, the steady-state solution is given as

$$\underset{t\to \infty }{\lim}I_{d}\left(t\right)≅\frac{W}{L}\mu_{0}C_{i}V_{d}a\tau \prime$$

The above result indicates that the trapped carriers and the current are generated only by the free carriers in equilibrium.

## 5. Discussion: Limitation and Data Causality Analysis

### 5.1. Limitations of Quantification of the Effects of Traps for the Hysteresis

**Figure 12:**
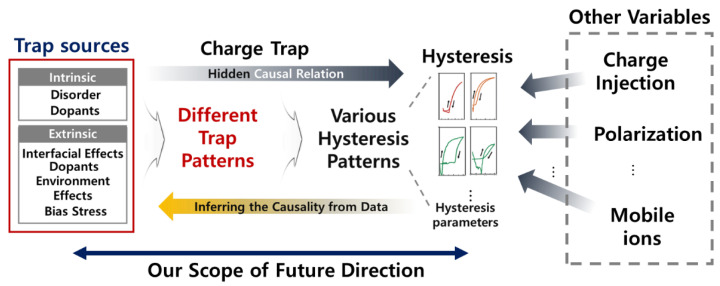
Scheme of limitations of quantification of causality between charge traps and hysteresis in OFETs.

In the previous sections, we have discussed the effect on the hysteresis in OFETs by charge traps, which are created due to various physical and chemical factors. However, we still face limitations in quantitatively determining how these traps affect the various factors of hysteresis. This is because there are many other factors apart from traps that affect hysteresis in OFETs, and the traps themselves are produced by various sources, as shown in Figure 12. Additionally, it is difficult to individually control the influencing factors through experiments, and it is also difficult to determine the uncontrollable or hidden factors that depend on time [29]. Therefore, it is difficult to determine the corresponding sources that produce the patterns of traps and their effect on the hysteresis [19]. Furthermore, it is difficult to classify the trap patterns due to limitations in the physical observation capabilities. 

However, identifying the quantitative effect of the trap on hysteresis and the cause of the trap can significantly help improve the performance of the OFETs by appropriately controlling the main variables. As explained in Section 3.2, it was observed from the ($I_{d}$, $V_{g}$) transfer curve that the factors affecting the traps occurring at the interface were the subthreshold swing and dielectric capacity. Therefore, it is essential to measure the appropriate hysteresis parameters through the ($I_{d}$, $V_{g}$) transfer curve and determine how these values change based on the degree of traps for the analysis of the correlation and causal relationship between the hysteresis and traps [19,32]. In the following subsection, to quantitatively analyze the relationship between these complex charge traps and hysteresis, we present data analysis techniques that are crucial in the field of machine learning (ML), beyond the physical/chemical approaches through existing experiments. These techniques help to quantitatively/statistically determine the effects of charge traps on hysteresis based on the experimentally obtained data.

### 5.2. Inferring the Causality from Data

**Figure 13:**
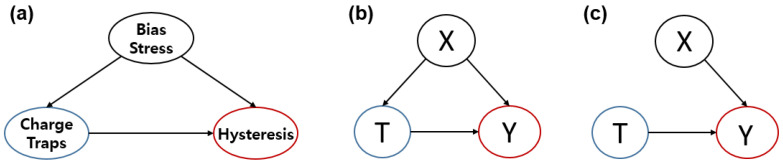
(**a**) Example of causal structure of hysteresis: the bias stress is a common cause of charge traps and the hysteresis, (**b**) Structure of substituting the nodes of (**a**) with variables X (covariate), T (treatment), and Y (result). (**c**) Causal structure when the treatment assignment mechanism is ignorable. Notably, this means there is no arrow from X to T and thus no confounding.

The technology used to estimate causal relations through data is rapidly developing due to the rapid increase in data and the development of various ML techniques that can efficiently process the data [78,79,80,81,82]. Causality is the relationship between an effect and its cause. Applying causality to data mining and ML domains is difficult since we must differentiate between causation and correlation, as stated by the well-known phrase: “**correlation is not causation**”. Typically, correlation and causation are different because a causal relationship can exist when there is a strong correlation between events, but the fact that two events occur sequentially and always occur together does not imply a causal relationship [79]. For example, consider the data in Figure 13. 

First, in Figure 13a, the bias stress affecting hysteresis and the causal structure of the charge trap are expressed through a simple graph. As discussed in the previous section, bias stress applied to OFETs can cause charge traps, but it can also cause hysteresis. For example, the bias stress causes a shift in the threshold voltage, a decrease in charge mobility, and an increase in the subthreshold slope, and hysteresis is sometimes observed. Therefore, in Figure 13a, bias stress is a factor that can affect both the charge trap and hysteresis, so it is marked with an arrow pointing to the two nodes in the graph. (The graph of this cause-and-effect relationship is called a “causal structure.”) What can be observed from this graph is that the charge trap affects hysteresis, but hysteresis is not necessarily generated only by the charge trap. In other words, there is a direct correlation between the charge trap and hysteresis, but causality cannot be determined yet. In order to understand this more formally, in Figure 13b, each node is expressed by substituting a variable. To see the causality of the charge trap for hysteresis, we set the variable X as a covariate (confounding), which represents a background variable (features); the variable T as the treatment, which is the action (manipulation or intervention), and the variable Y as the outcome, which is the result of the treatment. As in Figure 13c, if all effects from X to T are eliminated (i.e., other variables are assumed to be constant), we can accurately understand how Y is changed by T. This is the most basic approach to figuring out causality from data. This phenomenon typically occurs when the correlation between variables in the data is not clearly established or when there is a confounder variable that affects certain variables. However, by implementing recent deep representation learning methods, such as generative adversarial neural networks, confounders can be tuned by learning balanced representations for all covariates. Consequently, the conditional treatment assignments for learned representations are independent of the confounders. 

The causal inference framework can be classified into the following two categories: (i) **potential outcome framework** and (ii) **structural causal models (SCMs) framework**. The potential framework measures the effect of a specific treatment, and a SCM is a method of defining and analyzing the relationship between all the variables that can affect a certain variable. They are defined as follows:

(i) **Potential outcome framework:** Neyman and Rubin [83,84] proposed the potential outcome framework. In this framework, there are two possible outcomes for each unit if the treatments are binary values [85]. Here, T=0 indicates the control treatment, and T=1 is for the treated one. Consequently, there exist two potential outcomes, $Y\left(0\right)$ and $Y\left(1\right)$ by T=0 and T=1, respectively. In the experiment, we observe only one potential outcome corresponding to the assigned treatment T, which is called observed outcome Y. Otherwise, we refer to it as the counterfactual outcome. For the $i^{th}$ individual treatment $T_{i}$, the individual treatment effect (ITE) is defined by $\tau_{i}=Y_{i}\left(1\right)-Y_{i}\left(0\right).$ Thus, we can analyze the effect of treatment on the outcomes. For example, the average treatment effect (ATE) can be calculated as follows:
  $$ATE=E\left[\tau_{i}\right]=E\left[Y_{i}\left(1\right)-Y_{i}\left(0\right)\right]$$
  where $E\left[\tau_{i}\right]$ denotes the expectation of the random variable $\tau_{i}$ over an individual i. Therefore, the potential outcome framework determines the existence of a causality in which the outcomes can change based on the treatment.
(ii) **Structural causal models framework:** SCMs present an easy-to-see relationship of causality [86]. SCM is a method used to rearrange and analyze a structure using a causal graph as an equation [87]. Here, a causal graph, $G=\left(V,E\right)$, is a directed graph that represents the causal relationship between variables, where V denotes the node set and E denotes the edge set. Causal graphs are regarded as a special class of Bayesian networks, with edges representing causal relationships that satisfy the well-defined criterion of conditional independence. To better understand this concept, we consider only the modeling association without any causal modeling [84]. Consequently, if we model the data distribution $P\left(x_{1},\ldots ,x_{n}\right)$ for n data, $x_{1},\ldots ,x_{n},$ we can use the chain rule of probability to factorize any distribution, as follows:
  $$P\left(x_{1},x_{2},\ldots ,x_{n}\right)=P\left(x_{1}\right)\underset{i}{\prod }P(x_{i}|x_{i-1},\ldots ,x_{1})$$

**Figure 14:**
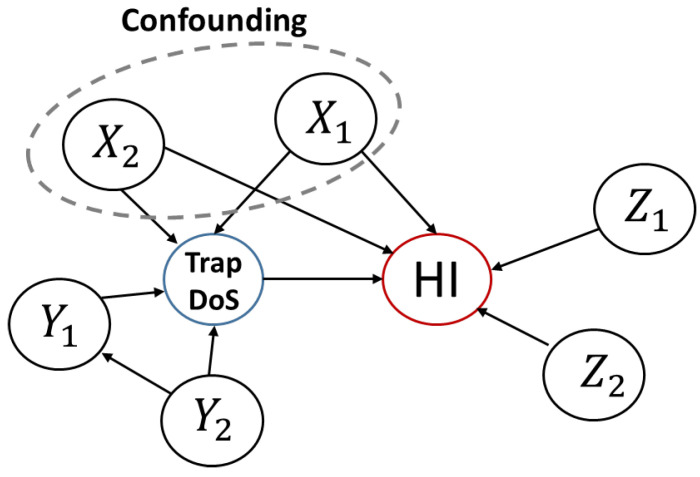
Examples of structural causal modeling using dependence of variables. Here, $X_{i}$ are common sources of the trap and hysteresis, $Y_{i}$ are the sources of the traps, and $Z_{i}$ are the sources of hysteresis, respectively.

If some of the local dependencies of the data are known a priori, we can construct a structured graph of the variables, which expresses the relationship between the effect of each variable in a highly simplified manner, as shown in Figure 14. The conditional independence contained in the causal graph of a given SCM provides sufficient information to determine whether a particular causal inference method meets the applicable criteria [87]. 

As described in the above two main approaches, causal inference is basically performed by a statistical approach from data obtained through experiments. For quantitative causal analysis of charge traps and hysteresis in OFETs, in general, the following three steps are considered:

1. **Defining variables and data preprocessing:** In this step, we first define the input variables and output variables considered in the experiment. For example, in the relationship between traps and hysteresis, trap sources can be input variables and the hysteresis index can be output variables. Next, an appropriate preprocessing step such as noise or outlier filtering is required for the values of the defined variables.
2. **Finding relationships by estimating joint distribution (or conditional distribution) from sampled data:** After the variables are well defined, it is necessary to statistically estimate the joint distribution or conditional distribution through the data sampling process to obtain the relationship between each variable. If it is difficult to accurately find the distribution through data, it can be approximated using well-known probability distribution models.
3. **Inferring causal relations by causal discovery methods:** Finally, based on the relationship between variables and data, an appropriate causal discovery algorithm is used to find a causal graph as shown in Figure 14, and based on this, the causality between each variable is analyzed.

In the causal analysis, several studies have been conducted on causal effect analysis or on causal relation discovery based on the given data using the two approaches presented above. In the next subsection, we present various algorithms for data-based causal relation discovery.

### 5.3. Causal Structure Discovery

As explained earlier, causal structure discovery involves learning the causal structure from the observational data [87]. Various algorithms have been proposed based on these data to identify the causal structure between the variables. For the causality analysis, many libraries and tools can be used [85]. The authors of [85] presented various discovery algorithms [88,89,90,91,92,93,94,95,96,97,98,99,100,101,102,103,104] such as double machine learning [92], meta-learners [93], and orthogonal learning [97,98], which have been supported by “EconML,” “CausalML,” “DoWhy,” and “CausalNex.” Additionally, several causal discovery methods, including graph inference, are supported by the “Causal Discovery Toolbox.” Recently, a novel method called “TIGRAMITE” was also developed for time series causal discovery. The authors [85] summarized the existing toolboxes in Table 4.

Consequently, in the relationship between the charge trap and hysteresis, a quantitative relationship between effects of the variable on the hysteresis behavior can be obtained by conditioning other parent parameters affecting the hysteresis and by applying the treatment operation to the control variable that causes the trap, as described in Figure 13c. For example, in OFETs, the relationship can be analyzed through the data obtained by fixing (setting as constants) other major factors affecting hysteresis and changing the factors directly affecting the trap. In addition, it may be necessary to find the causal relation that exists among the relationships between the factors influencing the trap and to find the causality of hysteresis by selecting variables that can be controlled. If a microscope is developed that can analyze the pattern of the charge trap, it could be used for causal analysis by applying visual causal feature learning [105,106] using the image information obtained through this method. Furthermore, it has been applied in the case of missing data [107]. 

## 6. Conclusions

In this work, we analyzed the effects of charge traps on the hysteresis phenomena in organic field-effect transistors. The characteristics and operating principles of the organic field-effect transistors were first examined, and the form of the drain current was analyzed corresponding to the gate–source voltage. In particular, we analyzed the charge trap, which is one of the main factors for the hysteresis phenomenon in organic field-effect transistors that appears during the gate-voltage sweep. Charge traps are caused by various intrinsic or extrinsic factors in organic field-effect transistors, and we analyzed the hysteresis caused by electron and hole traps. Various sources of charge traps were analyzed to get some insight into the effect of charge traps on hysteresis, and the models proposed thus far for trap density of state estimation were also reviewed. Additionally, a survey was conducted on the effect of hysteresis over time to determine the extent to which the threshold voltage changes and how the current-voltage transfer curve changes for the dynamic case where the trap changes over time. However, in organic field-effect transistors, various factors affect hysteresis apart from the charge trap, and the charge trap itself is produced by various sources. Thus, it is difficult to quantitatively determine how the patterns observed with hysteresis are affected by the trap using the existing experimental and simulation methods, which are considerably limited. This review proposes a quantitative causal relationship analysis based on machine learning technology for the data obtained from these physical experiments. Based on our literature survey and study on charge traps and hysteresis, we anticipate that machine learning-based data analysis offers a simple but robust charge-trap quantification by removing other factors causing hysteresis. We hope that this survey provides an important guideline for analyzing the causal relationship between inaccessible charge traps and hysteresis.

## Figures and Tables

**Table 1:** Three typical hysteresis mechanisms and their sources [19].

Mechanisms	Hysteresis Sources
**Effects near the semiconductor/dielectric interface**	Semiconductor/dielectric interface trap, injected charge from the semiconductor into the dielectric, slow reactions of mobile charge carriers, mobile ions in the semiconductor
**Bulk effects of the dielectric**	Polarization of the dielectric, mobile ions in the dielectric
**Charge injection from the gate**	Charge injection from the gate into the dielectric

**Table 2:** Summary of origins of charge traps in OFETs [32].

	Intrinsic	Extrinsic
**Trap Sources**	**Disorder** (Structural defects, chemical impurities, dynamic disorder) **Dopants** (Chemical impurities)	**Dopants** (Chemical impurities) **Interfacial Effects** (Semiconductor/dielectric interface, metal/semiconductor interface) **Environment Effects** (Water, oxygen, electromagnetic radiation) **Bias Stress Effect** (Bias stress)

**Table 3:** Summary of trap DoS approaches [64].

Paper	Trap DoS Estimation	Notations
Lang et al. [67]	$N\left(E\right)=\frac{C_{i}}{qa}{\left(\frac{dE_{a}}{dV_{g}}\right)}^{-1}$	$E_{a}$: activation energy a: accumulation layer thickness $V_{0}:$ interface potential p: volume hole density $\epsilon_{i},\;\epsilon_{s},\;\epsilon_{0}:$ dielectric constants $U_{g}:=\left|V_{g}-V_{FB}\right|$ $V_{FB}:$ flatband voltage l: thickness of the SiO_2_ gate dielectric $E_{a}^{\prime }\approx E_{V}-E_{F}-qV_{0}$ $E_{V}$: valence band edge energy $E_{F}$: Fermi energy
Horowitz et al. [68], Grunewald et al. [69]	$N\left(E\right)=\frac{1}{q}\frac{dp\left(V_{0}\right)}{dV_{0}}$	
Fortunato et al. [70]	$N\left(E\right)=\frac{\epsilon_{0}\epsilon_{s}}{2q}\frac{\partial^{2}}{\partial V_{0}^{2}}{\left(\frac{\epsilon_{i}}{\epsilon_{s}}\frac{U_{g}-V_{0}}{l}\right)}^{2}$	
Kalb et al. I [71]	$N\left(E\right)\approx \frac{d}{dE_{a}}\left[\frac{\epsilon_{0}\epsilon_{i}^{2}}{\epsilon_{s}l^{2}}U_{g}{\left(\frac{dE_{a}}{dU_{g}}\right)}^{-1}\right]\;$	
Kalb et al. II [64]	$N\left(E\right)\approx \frac{d}{dE_{a}^{\prime }}\left[\frac{\epsilon_{0}\epsilon_{i}^{2}}{\epsilon_{s}l^{2}}U_{g}{\left(\frac{dE_{a}^{\prime }}{dU_{g}}\right)}^{-1}\right]$	

**Table 4:** Toolbox for causal analysis [85].

Library	Feature	Algorithms
**DoWhy** [88]	ITE estimation	Propensity score matching [89] Stratification [90]
**EconML** [91]	ITE estimation and interpreter of the causal model	Double machine learning [92] Orthogonal random forests [94,95] Meta-learners [93] Deep instrumental variables
**Causal ML** [96]	ITE estimation	Meta-learners Uplift modeling [97,98]
**Causal Discovery Toolbox** [99]	Causal structure discovery	Graph inference Pairwise inference
**CausalNex**	Learning the causal structures and estimation of effects of potential interventions from data	Bayesian networks
**TIGRAMITE**	Time series datasets for causal discovery	PCMCI [100], generally [101], CMIknn [102], mediation class [103,104]
